# Co‐morbid sarcopenia and low bone mineral density in young paediatric cancer survivors

**DOI:** 10.1002/jcsm.13563

**Published:** 2024-08-20

**Authors:** Andres Marmol‐Perez, Esther Ubago‐Guisado, Jose J. Gil‐Cosano, Francisco J. Llorente‐Cantarero, Juan Francisco Pascual‐Gázquez, Manuel Muñoz‐Torres, Vicente Martinez‐Vizcaino, Kirsten K. Ness, Jonatan R. Ruiz, Luis Gracia‐Marco

**Affiliations:** ^1^ Department of Physical Education and Sports, Faculty of Sport Sciences, Sport and Health University Research Institute (iMUDS) University of Granada Granada Spain; ^2^ Department of Epidemiology and Cancer Control St. Jude Children's Research Hospital Memphis Tennessee USA; ^3^ Department of Communication and Education Loyola University Andalusia Seville Spain; ^4^ Maimonides Biomedical Research Institute of Cordoba (IMIBIC) Cordoba Spain; ^5^ Department of Specific Didactics, Faculty of Education University of Cordoba Cordoba Spain; ^6^ CIBEROBN, Biomedical Research Networking Center for Physiopathology of Obesity and Nutrition Carlos III Health Institute Madrid Spain; ^7^ Pediatric and Adolescent Hematology and Oncology Service, Pediatrics and Pediatric Surgery Clinical Management Unit Virgen de las Nieves University Hospital Granada Spain; ^8^ Biosanitary Research Institute Granada Spain; ^9^ Endocrinology and Nutrition Unit Hospital Universitario San Cecilio Granada Spain; ^10^ Department of Medicine, Faculty of Medicine University of Granada Granada Spain; ^11^ Centro de Investigación Biomédica en Red Fragilidad y Envejecimiento Saludable (CIBERfes) Instituto de Salud Carlos III Madrid Spain; ^12^ Health and Social Research Center Universidad de Castilla‐La Mancha Cuenca Spain

**Keywords:** Bone health, Cachexia, Childhood cancer, Exercise, Muscular health

## Abstract

**Background:**

Sarcopenia and low areal bone mineral density (aBMD) are prevalent musculoskeletal complications after paediatric cancer treatment. However, their relationship has not been examined in young paediatric cancers survivors. This study aimed to evaluate aBMD differences according to sarcopenia status and the risk of low aBMD *Z*‐score in young paediatric cancer survivors with sarcopenia confirmed/probable.

**Methods:**

This cross‐sectional study included 116 paediatric cancer survivors (12.1 ± 3.3 years old; 42.2% female). Handgrip strength was used to assessed muscle strength. Dual‐energy X‐ray absorptiometry estimated aBMD (g/cm^2^) and appendicular lean mass index (ALMI, kg/m^2^). ‘No sarcopenia’ was defined when muscle strength was >decile 2. ‘Sarcopenia probable’ was defined when muscle strength was ≤ decile 2 and ALMI Z‐score was > −1.5 standard deviation (SD). ‘Sarcopenia confirmed’ was defined when muscle strength was ≤ decile 2 and ALMI *Z*‐score ≤ −1.5 SD. Analysis of covariance and logistic regression, adjusted for time from treatment completion, radiotherapy exposure, calcium intake, and physical activity, was used to evaluate aBMD and estimate the odds ratios (ORs) of low aBMD (aBMD *Z*‐score < −1.0).

**Results:**

Survivors with sarcopenia confirmed had significantly lower aBMD than those without sarcopenia at total body (−1.2 [95% CI: −1.5 to −0.8] vs. 0.2 [−0.2 to 0.6], *P* < 0.001), lumbar spine (−0.7 [−1.1 to −0.3] vs. 0.4 [0.0 to 0.8], *P* < 0.001), total hip (−0.5 [−0.9 to −0.2] vs. 0.4 [0.1 to 0.8], *P* < 0.001), and femoral neck (−1.0 [−1.4 to −0.6] vs. 0.1 [−0.3 to 0.4], *P* = 0.001). Compared with survivors with sarcopenia probable, survivors with sarcopenia confirmed had significantly lower aBMD *Z*‐score at total body (−1.2 [−1.5 to −0.8] vs. −0.2 [−0.7 to 0.4], *P* = 0.009), total hip (−0.5 [−0.9 to −0.2] vs. 0.5 [−0.1 to 1.0], *P* = 0.010), and femoral neck (−1.0 [−1.4 to −0.6] vs. 0.1 [−0.5 to 0.7], *P* = 0.014). Survivors with sarcopenia confirmed were at higher risk of low aBMD *Z*‐score at the total body (OR: 6.91, 95% CI: 2.31–24.15), total hip (OR: 2.98, 1.02–9.54), and femoral neck (OR: 4.72, 1.72–14.19), than those without sarcopenia. Survivors with sarcopenia probable were at higher risk of low aBMD *Z*‐score at the total body (OR: 4.13, 1.04–17.60) than those without sarcopenia.

**Conclusions:**

Young paediatric cancer survivors with sarcopenia present higher risk of low aBMD. Resistance training‐based interventions designed to mitigate osteosarcopenia in this population should be implemented at early stages.

## Introduction

Paediatric cancer survival has significantly increased over the past 60 years reaching current 5‐year survival rates of 85% in children and 87% in adolescents.^1^ However, required treatments to cure cancer at such a young age increase the risk of later health‐related complications.^2^ Low areal bone mineral density (aBMD), defined by age‐, sex‐ and race‐specific aBMD *Z*‐score < T−1.0 standard deviation (SD), has been reported in up to two‐thirds of survivors.^3^ Early exposure to DNA damaging agents during childhood, during a vital period of active skeletal growth, decreases bone formation and increases bone resorption affecting bone development.^4^, ^5^ Moreover, these treatments not only interfere with bone health but also impact lean muscle mass and function.^6^ Paediatric cancer survivors present these limitations (hereafter referred to as sarcopenia)^7^ due to myofibrillary atrophy caused by degradation of myosin heavy chain and decrease in myosin synthesis death.^8^

Sarcopenia is currently considered a public health burden not only during adulthood,^9^ but also during childhood.^10^ It has been associated with a noteworthy vulnerability of adverse health outcomes following paediatric cancer treatment, including death.^11^ A previous study identified that sarcopenic adults had a four‐fold higher risk of having osteoporosis compared with non‐sarcopenic individuals.^12^ In adult paediatric cancer survivors, sarcopenia, pre‐frailty, and frailty (including low aBMD) have been reported to coexist at a mean age of 33 years.^13^ However, the literature depicting the associations of sarcopenia and low aBMD in this population is still scarce. Whether sarcopenia is associated with low aBMD right after paediatric cancer treatment completion, its detection could help survivors to be screened for low aBMD. This is relevant since sarcopenia diagnosis could anticipate further decline in aBMD, which is more exacerbated than in healthy population.^3^

The aims of this study were (i) to evaluate aBMD differences according to sarcopenia status and (ii) to examine the risk of low aBMD *Z*‐score in young paediatric cancer survivors with sarcopenia confirmed/probable. We hypothesized that survivors with sarcopenia would significantly present lower aBMD *Z*‐score and higher risk of low aBMD *Z*‐score than those without sarcopenia.

## Methods

### Study design and participants

This cross‐sectional study included 116 young paediatric cancer survivors (12.1 ± 3.3 years old; 42% female) from the iBoneFIT project framework. A detailed description of the study protocol has been published elsewhere.^14^, ^15^ Briefly, iBoneFIT is a multicenter parallel group randomized controlled trial designed to examine the effect of a 9‐month online exercise program on bone health in young paediatric cancer survivors.^14^ Survivors were recruited from the Units of Paediatric Oncology and Haematology of the ‘Virgen de las Nieves’ (Granada) and ‘Reina Sofia’ (Cordoba) University Hospitals. Inclusion criteria were one or more‐year survivors aged 6 to 18 years, not currently receiving treatments for cancer, at previous exposure to radiotherapy and/or chemotherapy. All measurements were conducted during in two waves due to COVID‐19 restrictions: first, from October to February 2020/2021; and second, from December to March 2021/2022. All parents and survivors provided written informed consent and assent before entering the trial, respectively. The iBoneFIT project was approved by the Ethics Committee on Human Research of Regional Government of Andalusia (Reference: 4500, December 2019), followed the ethical guidelines of the Declaration of Helsinki (revised version 2013), and the randomized controlled trial was registered (https://www.isrctn.com/ISRCTN61195625). This study was reported according to the Strengthening the Reporting of Observational Studies in Epidemiology (STROBE) checklist (Table S1). Although we recruited 116 young paediatric cancer survivors in total, sample size slightly varies for some variables due to missing data (i.e., some survivors were unable to perform some of the tests, were afraid of being scanned using Dual‐energy X‐ray absorptiometry [DXA] or declined a particular test during their assessment).

### Anthropometry and somatic maturity

Body mass (kg) was evaluated with an electronic scale (SECA 861, Hamburg, Germany) with an accuracy of 100 g. Stature (cm) was assessed using a precision stadiometer (SECA 225, Hamburg, Germany) to the nearest 0.1 cm. Body mass index was calculated as body mass (kg)/stature (m^2^). Additionally, age‐ and sex‐specific body mass index *Z*‐score and categories were calculated using international reference data for paediatric population.^16^ Somatic maturity was measured using the prediction of years before or after peak height velocity using validated algorithms for boys and girls.^17^

### Clinical data

Medical records were used to retrieve information regarding diagnosis, time from treatment completion to baseline data collection and treatment exposures (radiotherapy, chemotherapy and/or surgery, alone or in combination). Time from treatment completion (years) was treated as a continuous variable and treatment exposure as a dichotomous variable, radiotherapy (yes/no). Daily calcium (mg) intake was estimated by a validated specific food‐frequency questionnaire.^18^

### Physical activity

Tri‐axial ActiGraph wGT3x‐BT accelerometers (ActiGraph GT3X, Pensacola, FL, USA) were used to measure total physical activity (min/day) for seven consecutive days (24 hours/day). Survivors were instructed to wear devices on the non‐dominant wrist except for water activities. Accelerometers were initialized at a sampling frequency of 90 Hz, and raw data were processed using the GGIR R open‐source package version 2.8–2. Euclidean Norm of the raw acceleration minus one G with negative values rounded to zero (ENMO) was calculated, as well as the angle of the z‐axis of the device to estimate physical activity and sleep parameters.^19^ Non‐wear time was detected based on the standard deviation of the raw accelerations recorded in the three accelerometer axes as described elsewhere,^20^ and then imputed by means of the acceleration in the rest of the days at the same time window. Appropriate thresholds were used to identify physical activity intensities (i.e., Moderate‐to‐vigorous physical activity: 200 mg and light physical activity: 35–200 mg).^21^ We considered a day valid when (1) the accelerometer registered at least 23 h/day and (2) survivors wore the accelerometers on at least 16 hours/day since in this study the accelerometers were worn at both day and night.^22^ Survivors having at least one valid day were included (sensibility analyses showed similar results when compared with including participants having at least three valid weekdays and one weekend day). Total physical activity was calculated as the sum of daily average moderate‐to‐vigorous physical activity and light physical activity (mean of all 7 days).

### Dual‐energy X‐ray absorptiometry

Survivors were evaluated using a single DXA scanner (Hologic Series Discovery QDR, Bedford, MA, USA) and analysed by APEX software (version 4.0.2). The device was calibrated each day using a lumbar spine phantom. Survivors were asked to remain still and scanned in the supine position according to the ISCD.^23^ Three regions were analysed (total body, lumbar spine and right hip) to characterize aBMD (g/cm^2^) and bone mineral content (BMC, g) of total body (less head), lumbar spine (mean of L1–L4), total hip and femoral neck. A total body scan was used to obtain lean mass (kg) [body mass – (fat mass + bone mass)] of the arms and legs (appendicular lean mass), and of total body, and the trunk. Appendicular lean mass index (ALMI, kg/m^2^) was calculated by dividing appendicular lean mass by stature. A single trained researcher analysed all DXA scans. DXA coefficient of variation in paediatric population ranges between 1.0 and 2.9%, depending on the region.^24^

Using international reference data from the Bone Mineral Density in Childhood Study,^25^ age‐, sex‐ and race‐specific aBMD and BMC *Z*‐score at total body, lumbar spine, total hip and femoral neck were calculated for all analyses. Survivors with aBMD or BMC *Z*‐score ≤ −1.0 SD were classified with low aBMD or BMC, respectively.^3^ We used the same database to calculate age‐, sex‐ and race‐specific ALMI *Z*‐score for analyses.^26^ Survivors with ALMI ≤ −1.5 SD were classified with low ALMI as previous studies have conducted.^11^

### Muscle strength

Handgrip (upper‐body muscle strength) was evaluated with a dynamometer (TKK 5101 Grip D, Takei, Tokyo, Japan). This is a valid (intraclass correlation coefficients [ICC] 0.73–0.91), and reproducible test (ICC, 0.91–0.93).^27^ Survivors, keeping the arm straight, squeezed the dynamometer during 5 s twice by each hand and the best scores in kilograms were averaged.

To get an appropriate insight into the status of muscle strength in our sample, performance on each test was compared with updated age‐ and sex‐specific reference values of healthy young population based on nearly eight million test results from 34 countries gathered by the FitBack network.^28^ Muscle strength deficits were identified as ≤ decile 2 following previous sex‐and age‐specific percentiles definitions of fitness deficits first published by Blair et al.^29^

### Sarcopenia

**Figure 1:**
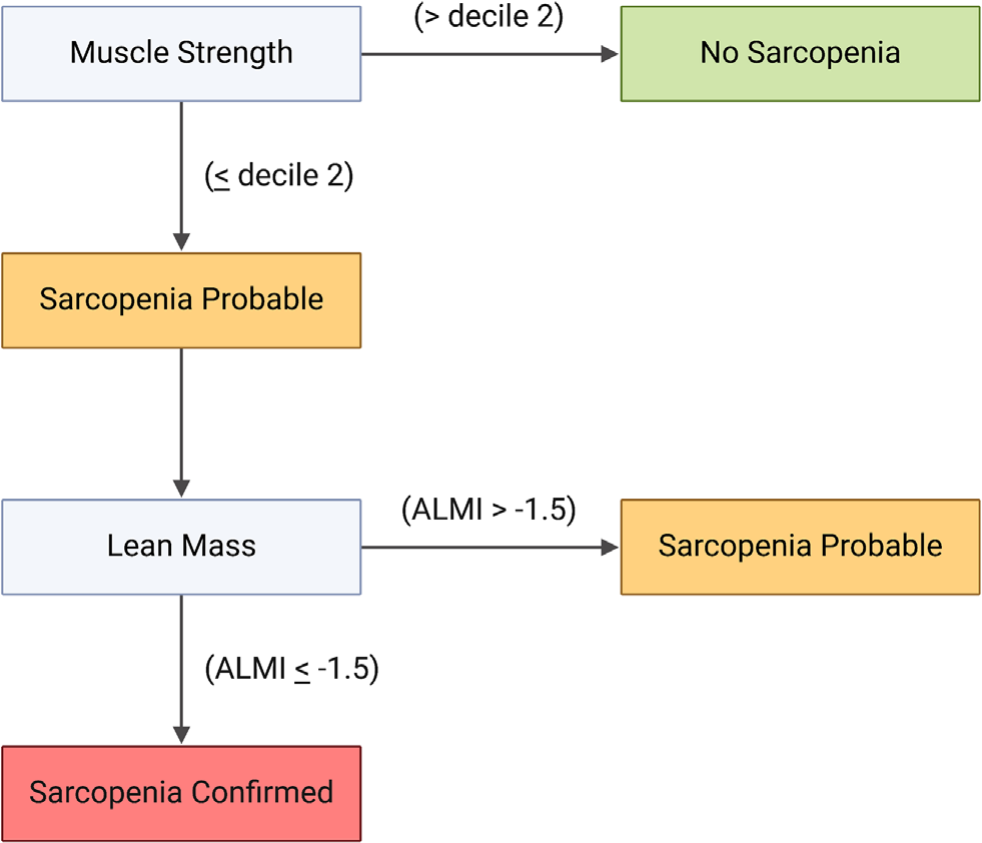
Sarcopenia status classification algorithm for identifying subjects with no sarcopenia, sarcopenia probable or sarcopenia confirmed (following the criteria of the sarcopenia definition stated by the EWGSOP2^30^). Muscle strength was compared with age‐ and sex‐specific reference values of healthy young population by the FitBack network.^28^ Using international reference data from the Bone Mineral Density in Childhood Study,^25^ age‐ and sex‐specific ALMI *Z*‐score was calculated. ALMI, appendicular lean mass index.

Sarcopenia status definition was followed according to the EWGSOP2 (Figure 1),^30^ following previous reports in this population.^13^ ‘No sarcopenia’ was defined when muscle strength was > decile 2. ‘Sarcopenia probable’ was defined when muscle strength was ≤ decile 2 and ALMI *Z*‐score was > −1.5 standard deviation (SD). ‘Sarcopenia confirmed’ was defined when muscle strength was ≤ decile 2 and ALMI *Z*‐score ≤ −1.5 SD. Given that the cut‐off points of the EWGSOP2 were based on adults, we compared muscle strength and ALMI with international reference data of healthy young population, as previously mentioned.

### Statistical analysis

Variable distributions were checked and verified using skewness and kurtosis, Kolmogorov–Smirnov test, visual check of histograms, Q‐Q and box plots. Descriptive data were reported as mean and standard deviation (SD) or as frequencies (percentages).

Analysis of covariance was used to test to evaluate aBMD score (outcome variable) according to sarcopenia status. Covariates included the time from treatment completion, radiotherapy exposure, calcium intake and total physical activity. To identify the minimum sufficient adjustment set (MSAS) for the differences in aBMD and BMC *Z*‐score according sarcopenia status, we built a theoretical causal diagram based on previous associations with muscle strength, lean mass and/or aBMD and BMC available in the scientific literature.^2^, ^31^, ^32^ We used the online tool DAGitty to construct a directed acyclic graph (DAG).^33^ The covariates age, sex, time from treatment completion, radiotherapy exposure, calcium intake, and physical activity were identified as the MSAS (Figure S1). Binary logistic regression models were used to estimate the odds of low aBMD of survivors with sarcopenia confirmed/probable. The same analyses were repeated with BMC *Z*‐score as the outcome. Results are presented as odds ratios (ORs) with 95% confidence intervals (CIs). Statistical analyses were performed using the statistical software R version 4.0.3 (R Foundation for Statistical Computing), B coefficient was presented non‐standardized, and *P*‐values < 0.05 were considered statistically significant.

## Results

A total of 196 young paediatric cancer survivors were initially approached for participation. After inclusion/exclusion criteria screening, 116 were enrolled and included in this study (Figure S2).

### Participant characteristics

Descriptive characteristics of the sample are presented in Table 1. Survivors were a mean (SD) age of 12.1 (3.3) years, 42.2% were female and the majority was diagnosed with blood cancers (60.9%). Table 2 shows that more than one‐third of survivors presented sarcopenia confirmed (37.9%) and that proportion was higher in males than females (43.3% vs. 30.6%, respectively). Males also presented higher proportions of low aBMD and BMC *Z*‐score than females at all sites, except for femoral neck BMC *Z*‐score (63.3% vs. 52.2%, respectively).

**Table 1:** Descriptive characteristics of the survivors included in the study

Characteristics	Total	*N*	Females	*N*	Males	*N*
Sex (female/male, %)	42.2/57.8	116				
Age (years)	12.1 ± 3.3	116	12.2 ± 3.5	49	12.0 ± 3.2	67
Body mass (kg)	46.6 ± 18.0	116	45.2 ± 18.3	49	47.6 ± 17.9	67
Stature (cm)	147.5 ± 17.1	116	145.3 ± 16.0	49	149.0 ± 17.7	67
Body mass index *Z*‐score	0.9 ± 1.1	116	0.8 ± 1.1	49	1.0 ± 1.2	67
Body mass index (categories, %)						
Underweight	3.5	4	6.1	3	1.5	1
Normoweight	61.2	71	65.4	32	58.2	39
Overweight	20.7	24	16.3	8	23.9	16
Obesity	14.6	17	12.2	6	16.4	11
Years from peak height velocity	−0.8 ± 2.7	116	0.0 ± 2.9	49	−1.3 ± 2.5	67
Time from treatment completion (years)	5.0 ± 3.8	113	5.2 ± 4.1	48	4.9 ± 3.6	65
Radiotherapy exposure (yes/no, %)	27.6/72.4	116	24.5/75.5	49	29.8/70.2	67
Type of cancer (categories, %)						
Blood	60.9	70	59.2	29	62.1	41
Solid	39.1	45	40.8	20	37.9	25
Calcium intake (mg)	785.5 ± 437.2	116	702.2 ± 388.6	49	846.4 ± 463.0	67
Physical activity (min/day)	297.7 ± 84.0	110	298.1 ± 94.1	48	297.4 ± 76.0	62

Data are presented as mean ± SD or as frequencies (%), as indicated.

**Table 2:** Distribution of sarcopenia status, age‐, sex‐, and race‐specific low areal bone mineral density (aBMD) and bone mineral content (BMC) *Z*‐score

Characteristics	Total	*N*	Females	*N*	Males	*N*
Muscle strength deficits (%)	56.9	66	59.2	29	55.2	37
Low ALMI *Z*‐score (%)	53.5	62	49.0	24	56.7	38
Sarcopenia status (%)						
No sarcopenia	43.1	50	40.8	20	44.8	30
Sarcopenia probable	19.0	22	28.6	14	11.9	8
Sarcopenia confirmed	37.9	44	30.6	15	43.3	29
Low aBMD *Z*‐score (%)						
Total body (less head)	25.9	30	24.5	12	26.9	18
Lumbar spine	26.7	31	18.4	9	32.8	22
Total hip	20.0	22	14.3	7	22.4	15
Femoral neck	27.6	32	24.5	12	29.9	20
Low BMC *Z*‐score (%)						
Total body (less head)	31.9	37	26.5	13	35.8	24
Lumbar spine	28.5	33	24.5	12	31.3	21
Total hip	18.1	21	16.3	8	19.4	13
Femoral neck	56.9	66	63.3	31	52.2	35

Data are presented as mean ± SD or as frequencies (%), as indicated.

aBMD, areal bone mineral density; ALMI, appendicular lean mass index; BMC, bone mineral content.

### Differences in aBMD *Z*‐score at each site according to sarcopenia status

**Figure 2:**
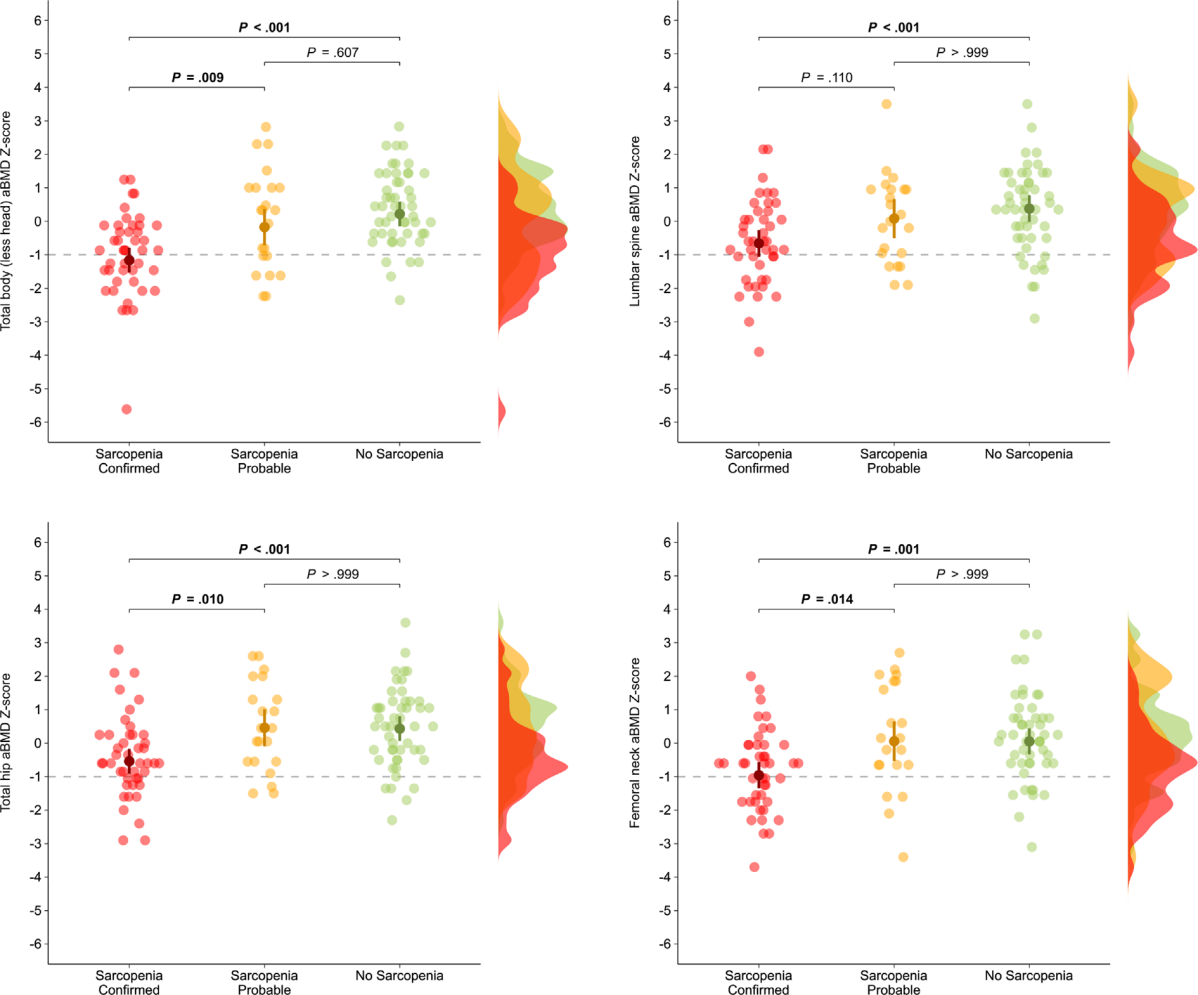
Differences in age‐, sex‐ and race‐specific areal bone mineral density (aBMD) *Z*‐score according to sarcopenia status in young paediatric cancer survivors. Data are presented as adjusted means and confidence intervals (95%). Half violin plots show the distribution within sarcopenia status. Significant differences (adjusted *P* < 0.05) between sarcopenia status are shown in bold by analysis of covariance. Analyses were adjusted for time from treatment completion to baseline evaluation (years) and radiotherapy exposure (yes/no). Grey dashed line indicates the cut‐off point for low areal bone mineral density according to van Atteveld et al. (2019).^3^

Figure 2 depicts that survivors with sarcopenia confirmed had significantly lower aBMD *Z*‐score than survivors with no sarcopenia at total body (−1.2 [95% CI: −1.5 to −0.8] vs. 0.2 [95% CI: −0.2 to 0.6], *P* < 0.001), lumbar spine (−0.7 [95% CI: −1.1 to −0.3] vs. 0.4 [95% CI: 0.0 to 0.8], *P* < 0.001), total hip (−0.5 [95% CI: −0.9 to −0.2] vs. 0.4 [95% CI: 0.1 to 0.8], *P* < 0.001), and femoral neck (−1.0 [95% CI: −1.4 to −0.6] vs. 0.1 [95% CI: −0.3 to 0.4], *P* = 0.001). In comparison with to survivors with sarcopenia probable, survivors with sarcopenia confirmed had significantly lower aBMD *Z*‐score at total body (−1.2 [95% CI: −1.5 to −0.8] vs. −0.2 [95% CI: −0.7 to 0.4], *P* = 0.009), total hip (−0.5 [95% CI: −0.9 to −0.2] vs. 0.5 [95% CI: −0.1 to 1.0], *P* = 0.010) and femoral neck (−1.0 [95% CI: −1.4 to −0.6] vs. 0.1 [95% CI: −0.5 to 0.7], *P* = 0.014). No differences were found between survivors with sarcopenia probable and no sarcopenia (*P* > 0.607). After adjusting for calcium intake and total physical activity, results were mostly similar (Figure S3). Likewise, when examining same analyses for BMC *Z*‐score, results were consistent (Figures S4 and S5).

### Risk of low aBMD *Z*‐score at each site according to sarcopenia status

The risk of low aBMD of survivors with sarcopenia confirmed/probable is presented in Table 3. Survivors with sarcopenia confirmed were at higher risk of low aBMD *Z*‐score at the total body (OR: 6.91, 95% CI: 2.31 to 24.15), total hip (OR: 2.98, 95% CI: 1.02 to 9.54) and femoral neck (OR: 4.72, 95% CI: 1.72 to 14.19), than those without sarcopenia. Survivors with sarcopenia probable were at higher risk of low aBMD *Z*‐score at the total body (OR: 4.13, 95% CI: 1.04 to 17.60), than those without sarcopenia. These results, controlled for time from treatment completion, radiotherapy exposure remained the same when calcium intake and total physical activity were additionally included in the models (Table S2). Findings were consistent when BMC *Z*‐score outcome variables were used (Tables S3 and S4).

**Table 3:** Odds ratios (95%) for low age‐, sex‐, and race‐specific areal bone mineral density (aBMD) *Z*‐score at each site according to sarcopenia status

	Normal aBMD (%)	Low aBMD (%)	OR	95% CI
Total body (less head)				
No sarcopenia	44 (89.8)	5 (10.2)	1.00	
Sarcopenia probable	16 (72.7)	6 (27.3)	**4.13**	**1.04 to 17.60**
Sarcopenia confirmed	23 (54.8)	19 (45.2)	**6.91**	**2.31 to 24.15**
Lumbar spine				
No sarcopenia	41 (83.7)	8 (16.3)	1.00	
Sarcopenia probable	16 (72.7)	6 (27.3)	2.09	0.60 to 7.17
Sarcopenia confirmed	27 (64.3)	15 (35.7)	2.56	0.95 to 7.27
Total hip				
No sarcopenia	43 (87.8)	6 (12.2)	1.00	
Sarcopenia probable	18 (85.7)	3 (14.3)	1.25	0.24 to 5.41
Sarcopenia confirmed	29 (69.0)	13 (31.0)	**2.98**	**1.02 to 9.54**
Femoral neck				
No sarcopenia	42 (85.7)	7 (14.3)	1.00	
Sarcopenia probable	17 (81.0)	4 (19.0)	1.51	0.35 to 5.94
Sarcopenia confirmed	23 (54.8)	19 (45.2)	**4.72**	**1.72 to 14.19**

Binary logistic regression (low aBMD identified as *Z*‐score < −1.0, and normal aBMD identified as *Z*‐score higher than −1.0) were used to estimate odds ratios with 95% confidence intervals. Adjusted models included time from treatment completion (years) and radiotherapy exposure (yes/no). Age‐, sex‐, and race‐specific aBMD *Z*‐score at each site are presented using international reference data from the Bone Mineral Density in Childhood Study. Bold values denote statistical significance (*P*‐values < 0.05).

aBMD, areal bone mineral density; CI, confidence interval; OR, odds ratio.

## Discussion

Over one‐third of young paediatric cancer survivors enrolled on this study presented sarcopenia confirmed and had significantly lower aBMD *Z*‐score than survivors with no sarcopenia or sarcopenia probable at all regions. Survivors with sarcopenia confirmed presented higher risk of low aBMD *Z*‐score at total body, total hip and femoral neck, than those without sarcopenia.

Previous reports^34^, ^35^, ^36^, ^37^ describing sarcopenia in young paediatric cancer survivors have not included functional outcomes. In our study, handgrip muscle strength in addition to ALMI via DXA were measured following the criteria of the sarcopenia definition stated by the EWGSOP2.^30^ The importance of including functional outcomes in the definition of sarcopenia is supported by our findings as not all survivors in our study with low ALMI had muscle strength deficits. Moreover, aBMD was not impaired at any site in survivors with low ALMI, but with normal muscle strength.

There are few reports describing concomitant sarcopenia and low aBMD in young paediatric cancer survivors. Previous studies have observed that muscle strength deficits are associated with low aBMD in young^38^ and adult paediatric cancer survivors,^31^ but very few have investigated whether having low ALMI in addition to muscle strength deficits (sarcopenia confirmed) would be associated with low aBMD even shortly after treatment completion. A study led by Guo et al.^39^ examined the link between sarcopenia status (measuring both lean mass and ankle dorsiflexion strength) and aBMD in 20 paediatric high‐risk neuroblastoma survivors (12.4 ± 1.6 years). In their study, survivors presented sarcopenia but not low aBMD after a median of 9 years from diagnosis (median of 2.8 years old at diagnosis). Their sample size was small and limited to high‐risk neuroblastoma survivors, whose treatment exposures likely differ from the exposures in our study population. Our results of coexisting geriatric symptoms are similar to a cohort study of 2,003 adult survivors of paediatric cancer that reported the coexistence of sarcopenia, pre‐frailty and frailty (including low aBMD) at a mean age of 33 years.^13^ Our findings suggest that sarcopenia and low aBMD coexist soon after completion of therapy. Resistance training‐based interventions designed to target both morbidities may prevent frailty and reduce the risk for fractures later in life.

### Limitations

Our results should be considered in the context of some potential limitations. Firstly, yet we present results controlling for major potential confounders identified through the DAG methodology (i.e., age, sex, time from treatment completion, radiotherapy exposure, physical activity and calcium intake), residual confounding cannot be disregarded. Secondly, reliance on aBMD systematically may systematically underestimate aBMD in shorter individuals because bone depth is not accounted for in DXA results. Thirdly, the survivors included in the present study were those who chose to participate in an exercise intervention to improve aBMD, and they may not be representative of all young paediatric cancer survivors.

### Public health implications

A myriad of studies have shown that young paediatric cancer survivors are at higher risk of muscle strength deficits, low lean mass and low aBMD.^3^, ^6^, ^38^ However, the interconnectedness between these premature complications have not been described together shortly after treatment completion. Given that screening for both age‐ and sex‐specific muscle strength deficits and low lean mass is clinically recommended, our study adds to the current literature that those with impairments should be referred not only for improving muscular weakness, but also to prevent further decline in bone mass. As sarcopenia and low aBMD are prevalent in adult paediatric cancer survivors,^13^ early sarcopenia identification and referral for rehabilitation are fundamental. These findings warrant further research based on well‐designed randomized controlled trials right after treatment completion.

## Conclusions

This study shows that sarcopenia is prevalent in young paediatric cancer survivors and is associated with higher risk of low aBMD *Z*‐score. These results suggest that sarcopenia detection in young cancer survivors at early stages after treatment completion could help survivors to be screened for low aBMD *Z*‐score. Further research is still needed to confirm these findings in larger cohort studies so that they could be included in surveillance guidelines.

## Funding

This study has been partially supported by the Spanish Ministry of Science and Innovation (Ref: PID2020‐117302RA‐I00), La Caixa Foundation (Ref: LCF/BQ/PR19/11700007) and the University of Granada Plan Propio de Investigación 2021‐Excellence Actions: Unit of Excellence on Exercise, Nutrition, and Health (UCEENS). AMP was also recipient of a predoctoral fellowship (FPU20/05530) by the Spanish Ministry of Education, Culture and Sport and EUG was supported by RYC2022‐038011‐I funding by MCIN/AEI/10.13039/501100011033 and ESF+.

## Conflict of interest

The authors declare that they do not have competing interests.

## Supporting information


**Figure S1.** Directed acyclic graph (DAG).
**Figure S2.** Flow chart.
**Figure S3.** Differences in age‐, sex‐ and race‐specific areal bone mineral density (aBMD) Z‐score according to sarcopenia status in young paediatric cancer survivors. Data are presented as adjusted means and confidence intervals (95%). Half violin plots show the distribution within sarcopenia status. Significant differences (adjusted P < .05) between sarcopenia status are shown in bold by analysis of covariance. Analyses were adjusted for time from treatment completion to baseline evaluation (years), radiotherapy exposure (yes/no), calcium intake (mg) and total physical activity (min/day). Grey dashed line indicates the cut‐off point for low areal bone mineral density according to van Atteveld et al. (2019)^1^
**Figure S4.** Differences in age‐, sex‐ and race‐specific bone mineral content (BMC) Z‐score according to sarcopenia status in young paediatric cancer survivors. Data are presented as adjusted means and confidence intervals (95%). Half violin plots show the distribution within sarcopenia status. Significant differences (adjusted P < .05) between sarcopenia status are shown in bold by analysis of covariance. Analyses were adjusted for time from treatment completion to baseline evaluation (years) and radiotherapy exposure (yes/no). Grey dashed line indicates the cut‐off point for low bone mineral content according to van Atteveld et al. (2019)^1^.
**Figure S5**. Differences in age‐, sex‐ and race‐specific bone mineral content (BMC) Z‐score according to sarcopenia status in young paediatric cancer survivors. Data are presented as adjusted means and confidence intervals (95%). Half violin plots show the distribution within sarcopenia status. Significant differences (adjusted P < .05) between sarcopenia status are shown in bold by analysis of covariance. Analyses were adjusted for time from treatment completion to baseline evaluation (years), radiotherapy exposure (yes/no), calcium intake (mg) and total physical activity (min/day). Grey dashed line indicates the cut‐off point for low bone mineral content according to van Atteveld et al. (2019)^1^.
**Table S1.** STROBE Statement. Checklist of items that should be included in reports of cross‐sectional studies.
**Table S2.** Odds ratios (95%) of low age‐, sex‐ and race‐specific areal bone mineral density (aBMD) Z‐score at each site according to sarcopenia status.
**Table S3.** Odds ratios (95%) of low age‐, sex‐ and race‐specific bone mineral content (BMC) Z‐score at each site according to sarcopenia status.
**Table S4.** Odds ratios (95%) of low age‐, sex‐ and race‐specific bone mineral content (BMC) Z‐score at each site according to sarcopenia status.
