# Supplementary material for: Co‐morbid sarcopenia and low bone mineral density in young paediatric cancer survivors
Source: J Cachexia Sarcopenia Muscle. 2024 Aug 20;15(5):2156–63. doi: 10.1002/jcsm.13563 (PMC11446677; doi:10.1002/jcsm.13563)
Supplement: Supplementary file 1 — Figure S1. Directed acyclic graph (DAG). Figure S2. Flow chart. Figure S3. Differences in age‐, sex‐ and race‐specific areal bone mineral density (aBMD) Z‐score according to sarcopenia status in young paediatric cancer survivors. Data are presented as adjusted means and confidence intervals (95%). Half violin plots show the distribution within sarcopenia status. Significant differences (adjusted P < .05) between sarcopenia status are shown in bold by analysis of covariance. Analyses were adjusted for time from treatment completion to baseline evaluation (years), radiotherapy exposure (yes/no), calcium intake (mg) and total physical activity (min/day). Grey dashed line indicates the cut‐off point for low areal bone mineral density according to van Atteveld et al. (2019)1 Figure S4. Differences in age‐, sex‐ and race‐specific bone mineral content (BMC) Z‐score according to sarcopenia status in young paediatric cancer survivors. Data are presented as adjusted means and confidence intervals (95%). Half violin plots show the distribution within sarcopenia status. Significant differences (adjusted P < .05) between sarcopenia status are shown in bold by analysis of covariance. Analyses were adjusted for time from treatment completion to baseline evaluation (years) and radiotherapy exposure (yes/no). Grey dashed line indicates the cut‐off point for low bone mineral content according to van Atteveld et al. (2019)1. Figure S5. Differences in age‐, sex‐ and race‐specific bone mineral content (BMC) Z‐score according to sarcopenia status in young paediatric cancer survivors. Data are presented as adjusted means and confidence intervals (95%). Half violin plots show the distribution within sarcopenia status. Significant differences (adjusted P < .05) between sarcopenia status are shown in bold by analysis of covariance. Analyses were adjusted for time from treatment completion to baseline evaluation (years), radiotherapy exposure (yes/no), calcium intake (mg) and total physical a [file JCSM-15-2156-s001.docx]

Comorbid Sarcopenia and Low Bone Mineral Density in Young Pediatric Cancer Survivors:

Andres Marmol-Perez, et al.

Supplementary material

**
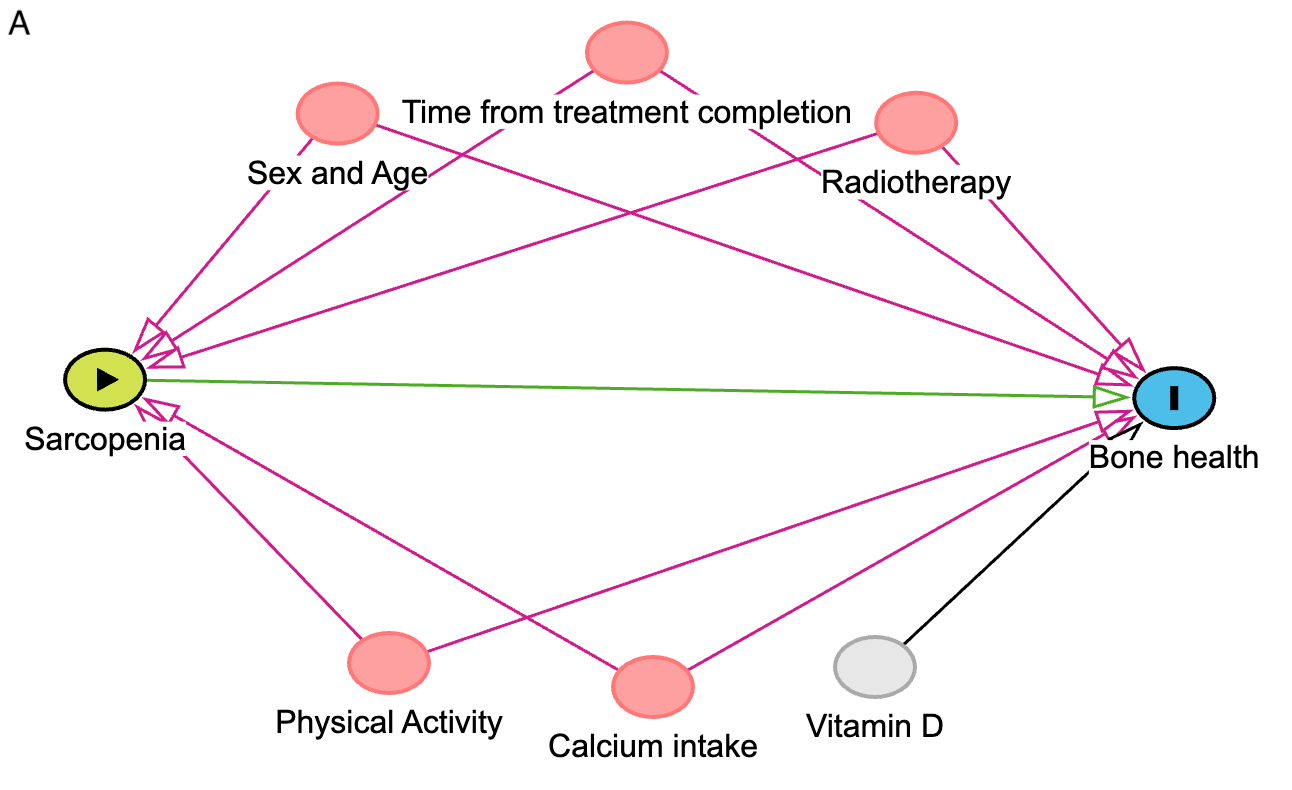
**

**
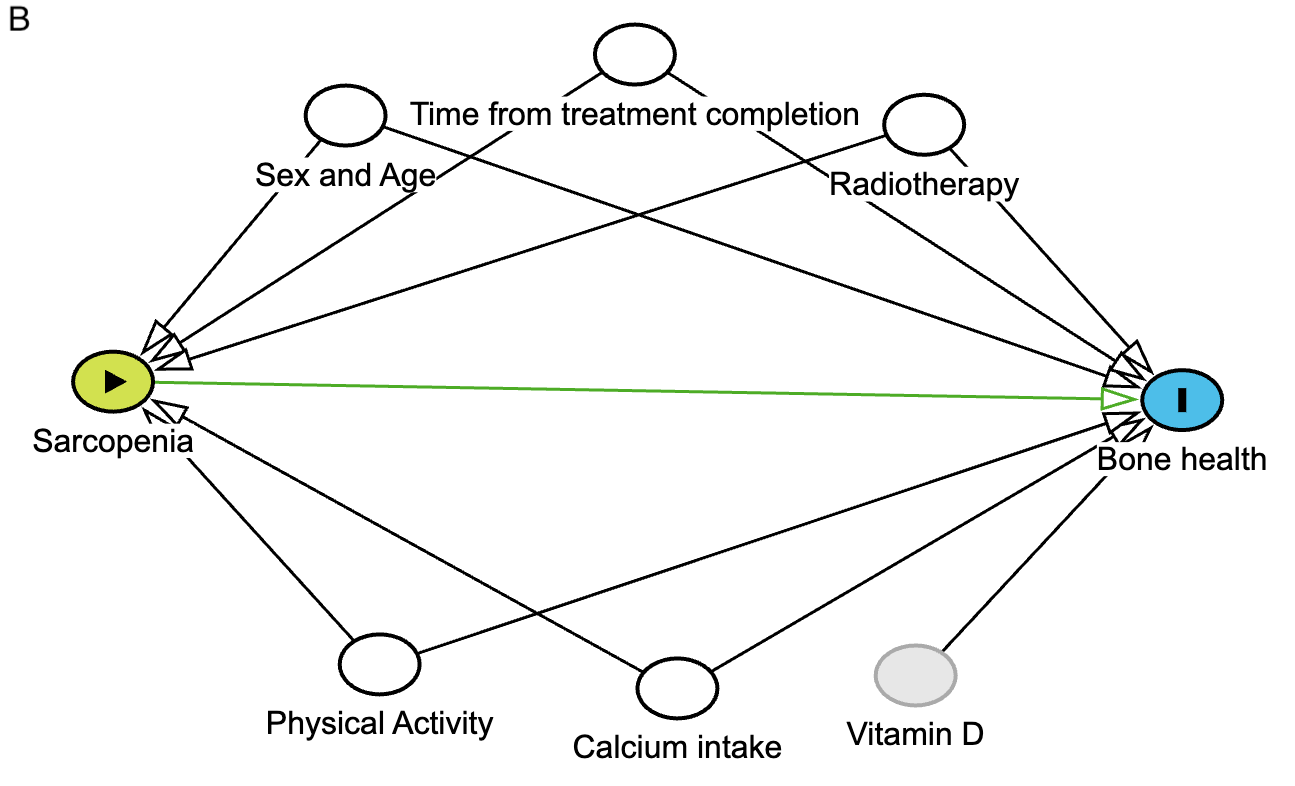
**

**Figure S1** Directed acyclic graph (DAG).

**Panel A** represents the DAG for the causal structure of the relationship between sarcopenia (exposure, green circle) and bone health (outcome, blue circle). Pink circles indicate ancestor variables of both the exposure and the outcome (sex, age, time from treatment completion, radiotherapy exposure, physical activity and calcium intake). Green arrows indicate "causal" paths, and pink arrows indicate biasing paths.

**Panel B** represent the DAG after adjusting for the minimum sufficient adjustment set for the total effect (i.e., sex, age, time from treatment completion, radiotherapy exposure, physical activity and calcium intake, now represented with white circles). Note that the biasing paths were completely closed (pink arrows became black arrows, suggesting the correct control for the relevant confounders), and that only the "causal" paths remained opened (both the direct path and the indirect paths, i.e., through mediators).





**Figure S2** Flow chart.

**
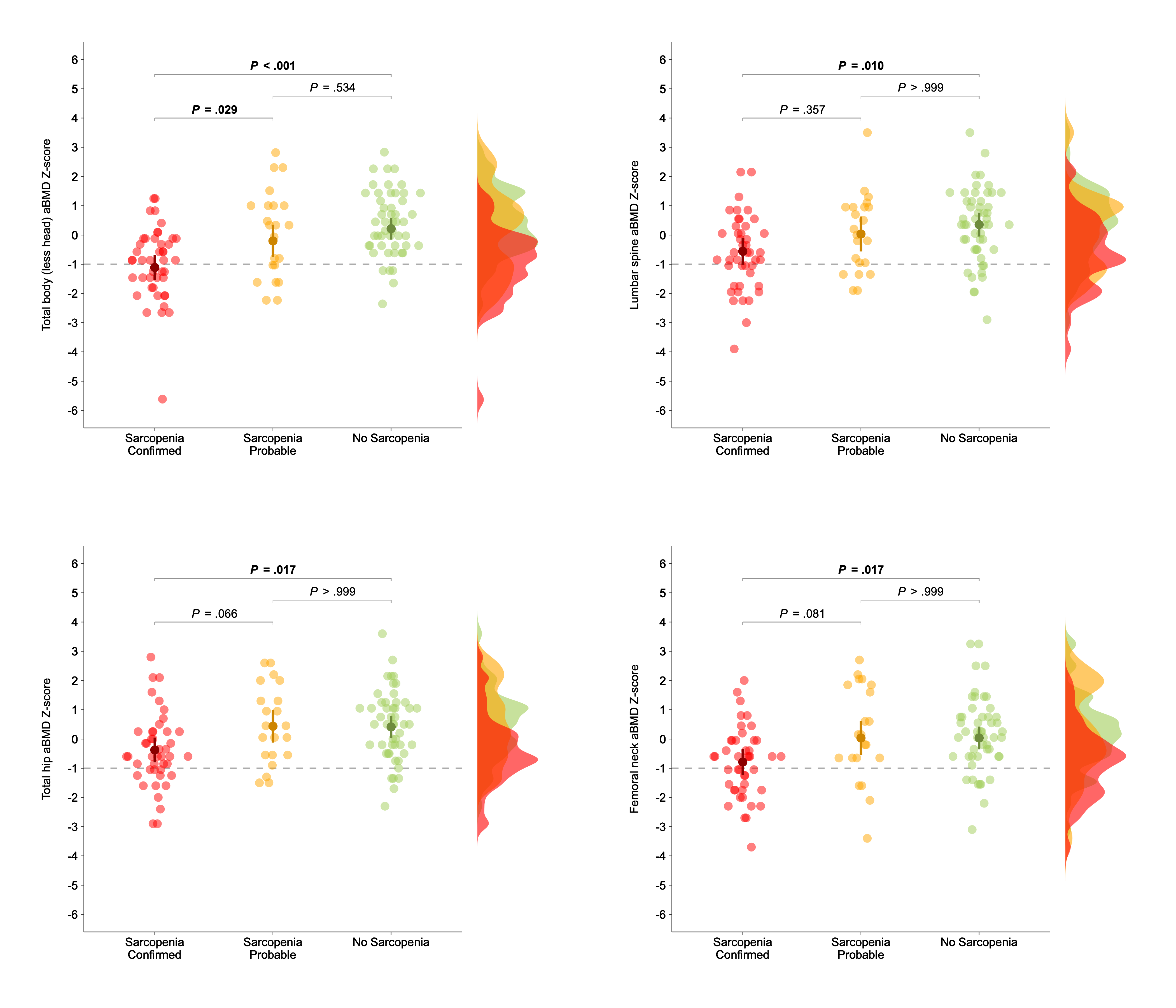
**

**Figure S3** Differences in age-, sex- and race-specific areal bone mineral density (aBMD) Z-score according to sarcopenia status in young paediatric cancer survivors. Data are presented as adjusted means and confidence intervals (95%). Half violin plots show the distribution within sarcopenia status. Significant differences (adjusted P < .05) between sarcopenia status are shown in bold by analysis of covariance. Analyses were adjusted for time from treatment completion to baseline evaluation (years), radiotherapy exposure (yes/no), calcium intake (mg) and total physical activity (min/day). Grey dashed line indicates the cut-off point for low areal bone mineral density according to van Atteveld et al. (2019) ^1^.


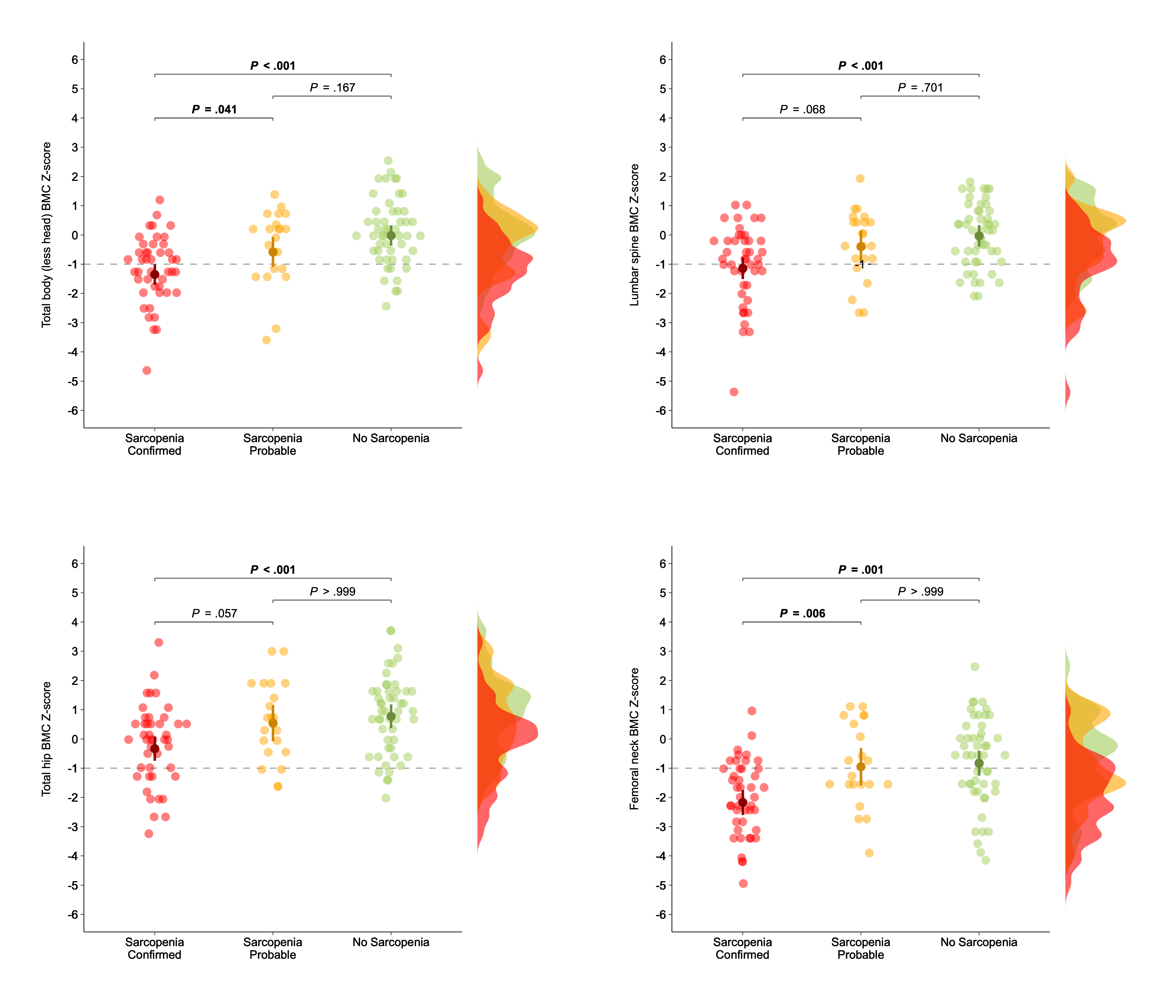


**Figure S4** Differences in age-, sex- and race-specific bone mineral content (BMC) Z-score according to sarcopenia status in young paediatric cancer survivors. Data are presented as adjusted means and confidence intervals (95%). Half violin plots show the distribution within sarcopenia status. Significant differences (adjusted P < .05) between sarcopenia status are shown in bold by analysis of covariance. Analyses were adjusted for time from treatment completion to baseline evaluation (years) and radiotherapy exposure (yes/no). Grey dashed line indicates the cut-off point for low bone mineral content according to van Atteveld et al. (2019) ^1^.


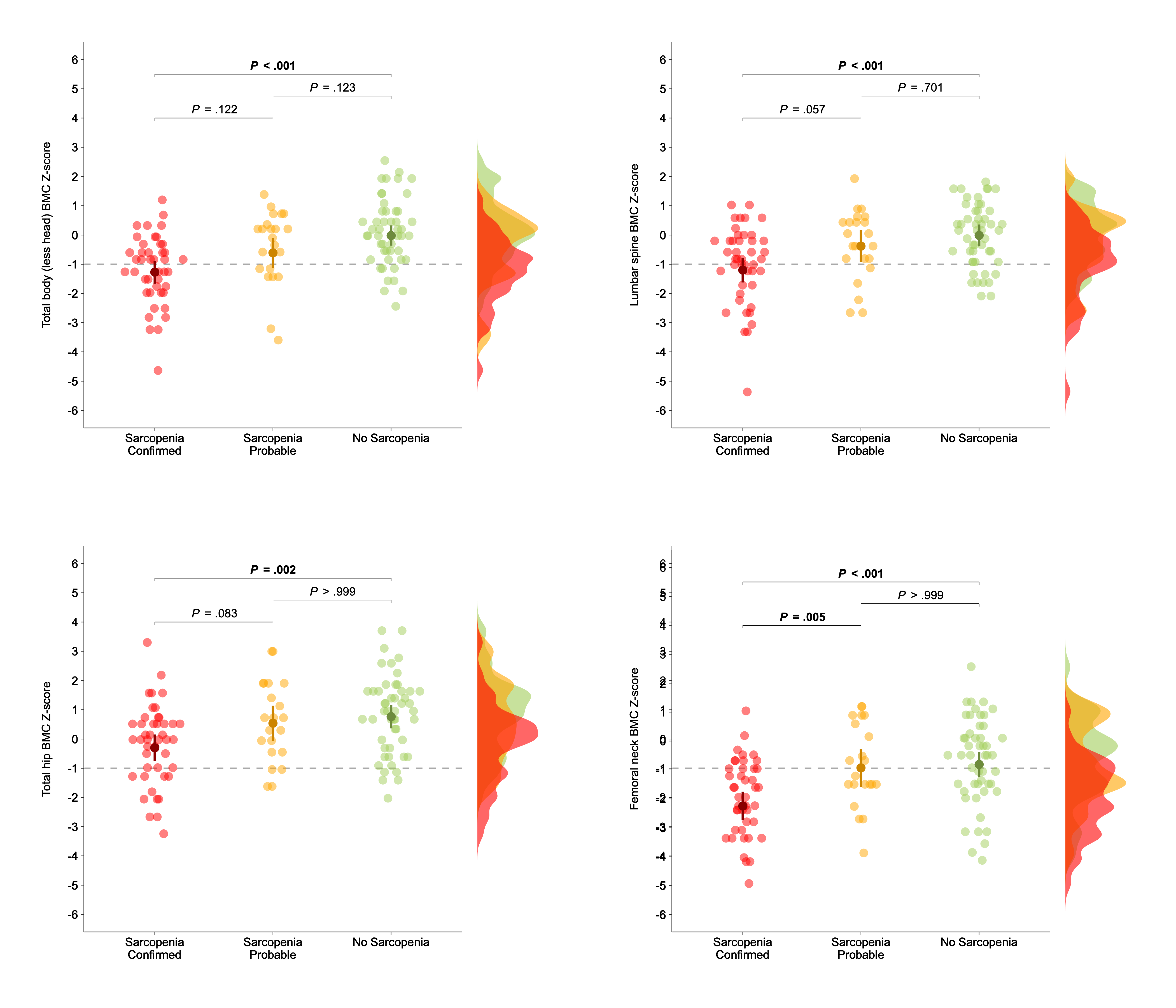


**Figure S5** Differences in age-, sex- and race-specific bone mineral content (BMC) Z-score according to sarcopenia status in young paediatric cancer survivors. Data are presented as adjusted means and confidence intervals (95%). Half violin plots show the distribution within sarcopenia status. Significant differences (adjusted *P* < .05) between sarcopenia status are shown in bold by analysis of covariance. Analyses were adjusted for time from treatment completion to baseline evaluation (years), radiotherapy exposure (yes/no), calcium intake (mg) and total physical activity (min/day). Grey dashed line indicates the cut-off point for low bone mineral content according to van Atteveld et al. (2019) ^1^.

**Table S1.** STROBE Statement. Checklist of items that should be included in reports of *cross-sectional studies.*

|  | **Item No** | **Recommendation** | **Page No** |
| --- | --- | --- | --- |
| **Title and abstract** | 1 | (*a*) Indicate the study’s design with a commonly used term in the title or the abstract | 1-2 |
|  |  | (*b*) Provide in the abstract an informative and balanced summary of what was done and what was found | 2 |
| **Introduction** | | | |
| Background/rationale | 2 | Explain the scientific background and rationale for the investigation being reported | 3 |
| Objectives | 3 | State specific objectives, including any prespecified hypotheses | 3 |
| **Methods** | | | |
| Study design | 4 | Present key elements of study design early in the paper | 3-4 |
| Setting | 5 | Describe the setting, locations, and relevant dates, including periods of recruitment, exposure, follow-up, and data collection | 3-4 |
| Participants | 6 | (*a*) Give the eligibility criteria, and the sources and methods of selection of participants | 3-4 |
| Variables | 7 | Clearly define all outcomes, exposures, predictors, potential confounders, and effect modifiers. Give diagnostic criteria, if applicable | 4-6 |
| Data sources/ measurement | 8 | For each variable of interest, give sources of data and details of methods of assessment (measurement). Describe comparability of assessment methods if there is more than one group | 4-6 |
| Bias | 9 | Describe any efforts to address potential sources of bias | 6-7 |
| Study size | 10 | Explain how the study size was arrived at | 3-4 |
| Quantitative variables | 11 | Explain how quantitative variables were handled in the analyses. If applicable, describe which groupings were chosen and why | 4-6 |
| Statistical methods | 12 | (*a*) Describe all statistical methods, including those used to control for confounding | 6-7 |
|  |  | (*b*) Describe any methods used to examine subgroups and interactions | 6-7 |
|  |  | (*c*) Explain how missing data were addressed | 6-7 |
|  |  | (*d*) If applicable, describe analytical methods taking account of sampling strategy | 6-7 |
|  |  | (*e*) Describe any sensitivity analyses | 6-7 |
| **Results** | | | |
| Participants | 13 | (a) Report numbers of individuals at each stage of study—eg numbers potentially eligible, examined for eligibility, confirmed eligible, included in the study, completing follow-up, and analysed | 7 |
|  |  | (b) Give reasons for non-participation at each stage | Figure S2 |
|  |  | (c) Consider use of a flow diagram | Figure S2 |
| Descriptive data | 14 | (a) Give characteristics of study participants (eg demographic, clinical, social) and information on exposures and potential confounders | Tables 1-2 and Figure S1 |
|  |  | (b) Indicate number of participants with missing data for each variable of interest | Tables 1-2 |
| Outcome data | 15 | Report numbers of outcome events or summary measures | Table 2 |
| Main results | 16 | (*a*) Give unadjusted estimates and, if applicable, confounder-adjusted estimates and their precision (eg, 95% confidence interval). Make clear which confounders were adjusted for and why they were included | Figure 2, Figures S3-5, Table 3 and Tables S3-5 |
|  |  | (*b*) Report category boundaries when continuous variables were categorized | Not applicable |
|  |  | (*c*) If relevant, consider translating estimates of relative risk into absolute risk for a meaningful time period | Not applicable |
| Other analyses | 17 | Report other analyses done—eg analyses of subgroups and interactions, and sensitivity analyses | 7-8 |
| **Discussion** | | | |
| Key results | 18 | Summarise key results with reference to study objectives | 8 |
| Limitations | 19 | Discuss limitations of the study, taking into account sources of potential bias or imprecision. Discuss both direction and magnitude of any potential bias | 9 |
| Interpretation | 20 | Give a cautious overall interpretation of results considering objectives, limitations, multiplicity of analyses, results from similar studies, and other relevant evidence | 9 |
| Generalisability | 21 | Discuss the generalisability (external validity) of the study results | 9-10 |
| **Other information** | | | |
| Funding | 22 | Give the source of funding and the role of the funders for the present study and, if applicable, for the original study on which the present article is based | 11 |

Note: An Explanation and Elaboration article discusses each checklist item and gives methodological background and published examples of transparent reporting. The STROBE checklist is best used in conjunction with this article (freely available on the Web sites of PLoS Medicine at http://www.plosmedicine.org/, Annals of Internal Medicine at http://www.annals.org/, and Epidemiology at http://www.epidem.com/). Information on the STROBE Initiative is available at [www.strobe-statement.org](http://www.strobe-statement.org).

**Table S2.** Odds ratios (95%) of low age-, sex- and race-specific areal bone mineral density (aBMD) Z-score at each site according to sarcopenia status.

|  | Normal aBMD (%) | Low aBMD (%) | OR | 95% CI |
| --- | --- | --- | --- | --- |
| Total body (less head) |  |  |  |  |
| No Sarcopenia | 44 (89.8) | 5 (10.2) | 1.00 |  |
| Sarcopenia Probable | 16 (72.7) | 6 (27.3) | **4.08** | **1.00 to 17.66** |
| Sarcopenia Confirmed | 20 (54.1) | 17 (45.9) | **6.54** | **2.02 to 24.43** |
| Lumbar spine |  |  |  |  |
| No Sarcopenia | 41 (83.7) | 8 (16.3) | 1.00 |  |
| Sarcopenia Probable | 16 (72.7) | 6 (27.3) | 2.25 | 0.63 to 7.94 |
| Sarcopenia Confirmed | 24 (64.9) | 13 (35.1) | 2.05 | 0.70 to 6.24 |
| Total hip |  |  |  |  |
| No Sarcopenia | 43 (87.8) | 6 (12.2) | 1.00 |  |
| Sarcopenia Probable | 18 (85.7) | 3 (14.3) | 1.24 | 0.23 to 5.58 |
| Sarcopenia Confirmed | 25 (67.6) | 12 (32.4) | 2.56 | 0.79 to 8.83 |
| Femoral neck |  |  |  |  |
| No Sarcopenia | 42 (85.7) | 7 (14.3) | 1.00 |  |
| Sarcopenia Probable | 17 (81.0) | 4 (19.0) | 1.49 | 0.33 to 6.09 |
| Sarcopenia Confirmed | 20 (54.1) | 17 (45.9) | **4.46** | **1.47 to 14.69** |

Notes: Binary logistic regression (low BMC identified as Z-score less than -1.0, and normal BMC identified as Z-score higher than -1.0) were used to estimate odds ratios with 95% confidence intervals. Adjusted models included time from treatment completion (years), radiotherapy exposure (yes/no), calcium intake (mg) and total physical activity (min/day). Age-, sex- and race-specific aBMD Z-score at each site are presented using international reference data from the Bone Mineral Density in Childhood Study ^2^. Bold values denote statistical significance (*P*-values < .05). Abbreviations: aBMD = areal bone mineral density; CI = confidence interval; OR = odds ratio.

**Table S3.** Odds ratios (95%) of low age-, sex- and race-specific bone mineral content (BMC) Z-score at each site according to sarcopenia status.

|  | Normal BMC (%) | Low BMC (%) | OR | 95% CI |
| --- | --- | --- | --- | --- |
| Total body (less head) |  |  |  |  |
| No Sarcopenia | 41 (83.7) | 8 (16.3) | 1.00 |  |
| Sarcopenia Probable | 15 (68.2) | 7 (31.8) | 2.81 | 0.83 to 9.72 |
| Sarcopenia Confirmed | 20 (47.6) | 22 (52.4) | **5.14** | **1.95 to 14.66** |
| Lumbar spine |  |  |  |  |
| No Sarcopenia | 40 (81.6) | 9 (18.4) | 1.00 |  |
| Sarcopenia Probable | 17 (77.3) | 5 (22.7) | 1.28 | 0.35 to 4.32 |
| Sarcopenia Confirmed | 24 (57.1) | 18 (42.9) | **3.49** | **1.35 to 9.66** |
| Total hip |  |  |  |  |
| No Sarcopenia | 43 (87.8) | 6 (12.2) | 1.00 |  |
| Sarcopenia Probable | 18 (85.7) | 3 (14.3) | 1.21 | 0.23 to 5.19 |
| Sarcopenia Confirmed | 30 (71.4) | 12 (28.6) | 2.79 | 0.95 to 9.01 |
| Femoral neck |  |  |  |  |
| No Sarcopenia | 28 (57.1) | 21 (42.9) | 1.00 |  |
| Sarcopenia Probable | 10 (47.6) | 11 (52.4) | 1.44 | 0.50 to 4.20 |
| Sarcopenia Confirmed | 8 (19.0) | 34 (81.0) | **6.60** | **2.47 to 19.68** |

Notes: Binary logistic regression (low BMC identified as Z-score less than -1.0, and normal BMC identified as Z-score higher than -1.0) were used to estimate odds ratios with 95% confidence intervals. Adjusted models included time from treatment completion (years) and radiotherapy exposure (yes/no). Age-, sex- and race-specific BMC Z-score at each site are presented using international reference data from the Bone Mineral Density in Childhood Study ^2^. Bold values denote statistical significance (*P*-values < .05). Abbreviations: aBMD = areal bone mineral density; CI = confidence interval; OR = odds ratio.

**Table S4.** Odds ratios (95%) of low age-, sex- and race-specific bone mineral content (BMC) Z-score at each site according to sarcopenia status.

|  | Normal BMC (%) | Low BMC (%) | OR | 95% CI |
| --- | --- | --- | --- | --- |
| Total body (less head) |  |  |  |  |
| No Sarcopenia | 41 (83.7) | 8 (16.3) | 1.00 |  |
| Sarcopenia Probable | 15 (68.2) | 7 (31.8) | 2.77 | 0.77 to 10.08 |
| Sarcopenia Confirmed | 17 (45.9) | 20 (54.1) | **4.83** | **1.66 to 15.13** |
| Lumbar spine |  |  |  |  |
| No Sarcopenia | 40 (81.6) | 9 (18.4) | 1.00 |  |
| Sarcopenia Probable | 17 (77.3) | 5 (22.7) | 1.28 | 0.34 to 4.36 |
| Sarcopenia Confirmed | 20 (54.1) | 17 (45.9) | **4.27** | **1.50 to 13.24** |
| Total hip |  |  |  |  |
| No Sarcopenia | 43 (87.8) | 6 (12.2) | 1.00 |  |
| Sarcopenia Probable | 18 (85.7) | 3 (14.3) | 1.02 | 0.18 to 4.68 |
| Sarcopenia Confirmed | 25 (67.6) | 12 (32.4) | 2.51 | 0.76 to 8.94 |
| Femoral neck |  |  |  |  |
| No Sarcopenia | 28 (57.1) | 21 (42.9) | 1.00 |  |
| Sarcopenia Probable | 10 (47.6) | 11 (52.4) | 1.44 | 0.49 to 4.28 |
| Sarcopenia Confirmed | 8 (21.6) | 29 (78.4) | **7.44** | **2.58 to 24.25** |

Notes: Binary logistic regression (low BMC identified as Z-score less than -1.0, and normal BMC identified as Z-score higher than -1.0) were used to estimate odds ratios with 95% confidence intervals. Adjusted models included time from treatment completion (years), radiotherapy exposure (yes/no), calcium intake (mg) and total physical activity (min/day). Age-, sex- and race-specific BMC Z-score at each site are presented using international reference data from the Bone Mineral Density in Childhood Study ^2^. Bold values denote statistical significance (*P*-values < .05). Abbreviations: aBMD = areal bone mineral density; CI = confidence interval; OR = odds ratio.

**References**

1. van Atteveld JE, Pluijm SMF, Ness KK, Hudson MM, Chemaitilly W, Kaste SC, et al. Prediction of Low and Very Low Bone Mineral Density Among Adult Survivors of Childhood Cancer. *J Clin Oncol* 2019;**37**:2217–25. https://doi.org/10.1200/JCO.18.01917.

2. Zemel BS, Kalkwarf HJ, Gilsanz V, Lappe JM, Oberfield S, Shepherd JA, et al. Revised Reference Curves for Bone Mineral Content and Areal Bone Mineral Density According to Age and Sex for Black and Non-Black Children: Results of the Bone Mineral Density in Childhood Study. *J Clin Endocrinol Metab* 2011;**96**:3160–9. https://doi.org/10.1210/JC.2011-1111.
